# Does Anxiety Affect Performance on Attention Task (digit span forward) on the MOCA Test? A clinical correlation study

**DOI:** 10.1093/geroni/igaa057.3243

**Published:** 2020-12-16

**Authors:** Rima Patel, Anil Nair

**Affiliations:** 1 The Alz Center, Burlington, Massachusetts, United States; 2 Alzheimer’s Disease Center, Quincy, Massachusetts, United States

## Abstract

It is unknown if anxiety affects performance on Digit span forward (DSF) in memory clinic patients. We performed a retrospective chart review of memory clinic patients in the south shore of Boston from 2010 to 2020. We correlated anxiety screen data (GAD7) to Digit Span Forward (DSF) scores obtained from the MoCA. As the data were not normal, we performed univariate analyses with Spearman correlation. A multivariate regression model estimated the relationship of DSF to covariates of GAD7, age, sex, and race. We hypothesized a negative correlation between anxiety levels scored by GAD7 and DSF. H0: Digit span forward DSF ~ GAD7+Age+Sex+Race. A chart review found 965 patients attending the memory clinic between 2010 to 2020 had analyzable data. 433 patients with available DSF and 737 had available GAD7. The patients were 58.7% female and 84.7% caucasian. The mean age was 70.1±14.4, DSF 0.8±0.4 and GAD 5.6±5.7. DSF correlated significantly to race (⍴=-0.25, p=<0.001), but not to gender (⍴=0.05, p=0.149), age (⍴=-0.04, p=0.3), or GAD7 (⍴=-0.018, p=0.71). There was no significant association of DSF to race, age, gender or GAD7 on the multivariate model. In memory clinic subjects there exists no correlation between anxiety levels scored by GAD7 and DSF performance.

